# Carcinoma of the Bladder*Abstract of clinical lecture given in the Long Island College Hospital.

**Published:** 1916-10

**Authors:** Henry H. Morton

**Affiliations:** Clinical Professor of Genito-Urinary Diseases in the Long Island College Hospital, Genito-Urinary Surgeon to Long Island College and King’s County Hospitals, and the Polhemus Memorial Clinic, Etc., Brooklyn, N. Y.


### Carcinoma of the Bladder.*

BY HENRY H. MORTON, M. D., CLINICAL PROFESSOR OF GENITO-URINARY DISEASES IN THE LONG ISLAND COLLEGE HOSPITAL, GENITO-URINARY SURGEON TO LONG ISLAND COLLEGE AND KING'S COUNTY HOSPITALS, AND THE POLHEMUS MEMORIAL CLINIC, ETC., BROOKLYN, N. Y.

Every bladder tumor in time becomes malignant and kills the patient by constantly recurring and continuing hemorrhage, or an ascending pyelo-nephritis.

There are no symptoms of bladder cancer till bleeding begins, at first a painless intermittent hematuria, which later becomes more or less constant.

The diagnosis can be made only by cystoscopic examination.

The earliest attempts at operation for bladder tumors consisted in simply twisting or scraping off the new growth, but recurrences were frequent. Later, they were removed by the actual cautery, to destroy the base. The results were a little better, but recurrences were the rule.

All operative attempts in cancer of the bladder have been discouraging, as very often the disease was spread, or the suprapubic wound failed to close.

According to the European clinicians the bladder should be opened only in—

1. Neoplasms involving the dome or front.
2. Budding neoplasms whose offshoot clog the neck of the bladder like a stopper in a bottle and cause retention of urine.
3. Neoplasms causing hematuria where hemorrhage makes intervention necessary.

When the tumor involves the floor or is around the ureteral orifice it is better to leave it alone.

In the final stages to make the patient a little more comfortable a suprapubic cystostomy with permanent fistula or a double nephrostomy may be done.

Fulguration with the Oudin current is the ideal treatment for non-malignant growths, but its use in malignant growths only serves to check hemorrhage or slightly retard the growth of the tumor.

At present the action of radium is too uncertain and not sufficiently worked out to be depended upon.

*Abstract of clinical lecture given in the Long Island College Hospital.

Removal of the tumor and resection of the bladder wall for one inch around the base promises to be of some benefit in bladder cancer provided it is done early.

The general practitioner should bear in mind that every case of hematuria demands an immediate cystoscopic examination, and if the hematuria is caused by bladder cancer, an early diagnosis with prompt removal affords at least a possibility of saving the patient's life.

H. V. Raycroft, M. D.

